# An Analysis of the Water-to-Ice Phase Transition Using Acoustic Plate Waves

**DOI:** 10.3390/s21030919

**Published:** 2021-01-29

**Authors:** Vladimir Anisimkin, Vladimir Kolesov, Anastasia Kuznetsova, Elizaveta Shamsutdinova, Iren Kuznetsova

**Affiliations:** Kotelnikov Institute of Radio Engineering and Electronics of RAS, 125009 Moscow, Russia; kvv@cplire.ru (V.K.); tigrnasya@yandex.ru (A.K.); Shes1996@bk.ru (E.S.); kuziren@yandex.ru (I.K.)

**Keywords:** acoustic wave, piezoelectric plate, liquid, ice, phase transition, acoustic attenuation

## Abstract

It is shown that, in spite of the wave radiation into the adjacent liquid, a large group of Lamb waves are able to propagate along piezoelectric plates (quartz, LiNbO_3_, LiTaO_3_) coated with a liquid layer (distilled water H_2_O). When the layer freezes, most of the group’s waves increase their losses, essentially forming an acoustic response towards water-to-ice transformation. Partial contributions to the responses originating from wave propagation, electro-mechanical transduction, and wave scattering were estimated and compared with the coupling constants, and the vertical displacements of the waves were calculated numerically at the water–plate and ice–plate interfaces. The maximum values of the responses (20–30 dB at 10–100 MHz) are attributed to the total water-to-ice transformation. Time variations in the responses at intermediate temperatures were interpreted in terms of a two-phase system containing both water and ice simultaneously. The results of the paper may turn out to be useful for some applications where the control of ice formation is an important problem (aircraft wings, ship bodies, car roads, etc.).

## 1. Introduction

Water is one of the most interesting liquids in nature. One of its peculiarities is the presence of the water-to-ice phase transition. This phase transition has been studied both theoretically and experimentally in a number of papers [1,2,3,4,5]. The influence of water impurities and the physical properties of water and solid substrates on the transition has been examined [6,7,8,9]. The water properties at critical and supercritical conditions have also been investigated [10,11,12]. Different experimental methods such as Raman spectroscopy [4,6,9], X-ray absorption spectroscopy [13], and high-pressure differential scanning calorimetry [14] have been applied for research in this area.

In general, the detection of water-to-ice transition and ice formation is an important problem for many applications, including aircraft, ships, roads, rotating elements, etc. [15]. In recent years, this problem has been solved using several experimental techniques, such as acoustic vibration [16,17], electro-optics [18], fiber optics [19], radio frequency [20], micro-mechanical sensors [21], and inductive devices [22]. Among all these techniques, the ultrasonic approach based on acoustic wave propagation in solids is considered to be one of the most attractive, as it is usually characterized by a fast response, high sensitivity, small size, and low energy consumption. For example, the ultrasonic technique has already been used for measuring ice thickness [23] and the water/ice phase transition using surface acoustic waves (SAWs) [24,25,26] and waves with a shear-horizontal polarization [27,28,29,30,31]. On the other hand, Lamb waves propagating in plates and possessing a variety of sensing properties have not yet been exploited for the same measurements, because it was thought that, having large normal displacement components, these waves would radiate into the adjacent medium (liquid) too strongly and attenuate along the propagation path too fast. Meanwhile, since many types of acoustic waves are now known in bulk substrates, layered structures, and piezoelectric plates [32,33,34,35,36,37,38], it could be expected that the ultrasonic detection of the liquid–solid phase transitions and ice formations may be essentially improved. Moreover, the influence of water–ice phase transitions on the acoustic wave properties may also be examined.

The goal of the present paper is to study new facilities of the ultrasonic technique based on different piezoelectric crystals and various acoustic plate waves, whose sensing properties may be varied with mode order n, plate thickness h, and acoustic wavelength λ.

## 2. Materials and Methods

**Figure 1:**
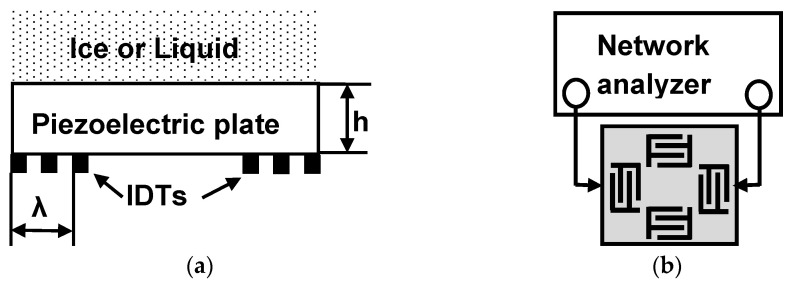
Schematic view of the test sample (**a**) and equipment (**b**).

The measurements were carried out at atmospheric pressure using delay lines (Figure 1) implemented on piezoelectric plates of Y,Z-LiNbO_3_ (Eugler angles 0°, 90°, 90°), Y,Z+ 90°-LiNbO_3_ (0°, 90°, 0°), ST,X-quartz (0°, 132.75°, 0°), ST,X+ 90°-quartz (0°, 132.75°, 90°), 360Y,X-LiTaO_3_ (0°, −54°, 0°), and 36°Y,X+ 90°-LiTaO_3_ (0°, −54°, 90°) (Crystal Technology Inc., West Chester, PA, USA). The plates had a 500 µm thickness with one grinded and one polished surface. The grinded surface (optical class Δ10) had an average horizontal and vertical roughness of 0.16 and 0.8 μm, respectively. The polished surface (optical class Δ14) had an average horizontal and vertical roughness of 0.01 and 0.05 μm, respectively. The grinded surface was coated with the test sample (water or ice), 600 mg in mass, deposited on the propagation path directly, without using a special cell. The absence of the cell avoids the ice sample falling off the plate surface at low temperatures, providing rigid ice/plate contact. The thickness of the ice layer was about 0.5 mm.

The polished surface of the plates contained two pairs of input and output interdigital transducers (IDTs) with periodical structures aligned perpendicular to each other in order to generate two families of Lamb waves in the same substrate. Each transducer was comprised of 40 finger electrodes followed with period *λ* = 300 μm patterned from 100 nm-thick Cr and 1000 nm-thick Al. The large number of electrodes provided a narrow (5%) transducer pass band and good frequency resolutions from the modes with close velocities *V_n_*. The value of the normalized plate thickness *h*/*λ* = 500 μm/300 μm = 1.67 (*h*-plate thickness, *λ*-wavelength, period of transducers) ensured a large variety of excited modes.

Acoustic delay lines were placed in a climatic chamber UC-20CE (NOSELAB ATS, Nova Milanese, Italy) with 20 L in volume and a variable temperature (*T*) from −60 °C to +150 °C with a step of <0.1 °C. The time of the temperature variation from one value to the other depended on the interval between them. For example, cooling from +20 °C to −15 °C took 1100 s. The temperature of the plate was additionally controlled using an iron–constantan thermocouple.

The insertion loss of the delay lines (transfer function *S*_12_) versus frequency *f* was measured with a network analyzer E5061B (Keysight, Santa Rosa, CA, USA) KEYSIGHT 5061B, operating in the amplitude–frequency format [39]. In order to avoid the influence of electro-magnetic leakage, the format *S*_12_ (*f*) was converted to the amplitude–time format *S*_12_ (*τ*), where the leakage was rejected using a proper gate start (just after leakage) and gate stop (just after the acoustic signal). Then, the time format *S*_12_ (*τ*) was converted back to the frequency format *S*_12_ (*f*) to obtain the measurements without leakage.

The procedure of the measurements was as follows: First, for each mode *n* the loss $S_{12}^{air}$ was measured in air at +20 °C, and the relevant frequency *f_n_* *= V_n_/λ*, where *V_n_* is the mode velocity and *λ* is the transducer period (the wavelength). Then, the same loss $S_{12}^{lq}$ was recorded after a test liquid (distilled water, 600 mg in mass) was deposited on the plate at +20 °C. Finally, the delay line was cooled from +20 to −15 °C and the loss $S_{12}^{ice}$ was measured at −15 °C, the point at which the liquid had completely transformed to ice. The sensitivity of the mode towards the liquid-to-ice phase transition (ice formation) was defined as $\Delta S_{12}=S_{12}^{ice}-S_{12}^{lq}$. Optimization of the modes, frequencies, and plate materials in the paper was accomplished in two steps. In the first step, the frequency of the most sensitive mode (the mode with the maximum response to icing) was found for each material. In the second step, different plate materials with the same thickness *h*/*λ* = 500 μm/300 μm = 1.67 were compared with one another, and the mode (frequency) with the largest ice response (maximum response to icing) was determined for this group of materials at a fixed plate thickness of *h*/*λ* = 1.67 for all of them. Different modes *n* and various plate materials (quartz, LiNbO_3_, LiTaO_3_) were compared with each other and the best mode/material combination was taken for detailed investigation.

In the beginning, the best combination was studied when the acoustic delay line with the test liquid was cooled without steps from +20 °C to −15 °C (liquid–ice transition) and then heated from −15 °C to +20 °C (ice–liquid transition). This procedure allowed the comparison of the final stages of the phase transitions for different plate materials. After that, the best combination was examined when the acoustic delay line with the test liquid was fluently cooled from +20 °C to −15 °C. In this case, the temperature changed in intervals of 5 °C with pauses of 60 s at each temperature. This procedure allowed the study of different steps of the transition processes at intermediate temperatures.

**Figure 2:**
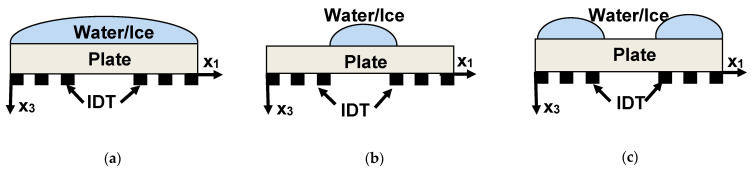
Delay line structures used for the analysis of the partial contributions to the Lamb wave water or ice response. (**a**) Water or ice is deposited on the entire surface of the plate, (**b**) surface of the plate between IDTs (**c**) surface of the plate over IDTs.

Detection mechanisms responsible for ice-sensing with Lamb waves were analyzed using the delay line structures shown in Figure 2a–c. The structure in Figure 2a contains a liquid or ice sample over the whole propagation path, including two regions opposite to transducers. The structure in Figure 2b confines a liquid or ice sample only between transducers. Finally, the structure in Figure 2c positions the same liquid/ice samples only on the regions opposite to transducers. For each structure, the insertion losses $S_{12}^{lq}$ (Figure 2a–c) and $S_{12}^{ice}$ (Figure 2a–c) are measured independently and presented analytically through their partial components as follows:

- For delay lines with water loadings:
  (1) $$S_{12}^{lq}\;(a)\;=\;2{TL}^{lq}\;+\;22.5\;\times \alpha^{lq}$$
  (2) $$S_{12}^{lq}\;(b)\;=\;2{TL}^{air}\;+\;2{St}^{lq}+\;10.5\;\times \;\alpha^{lq}$$
  (3) $$S_{12}^{lq}\;(c)\;=\;2{TL}^{air}\;+\;2{St}^{lq}\;+\;12\;\times \;\alpha^{lq}$$
- For delay lines with ice loadings:
  (4) $$S_{12}^{ice}\;(a)=2{TL}^{ice}+22.5\;\times \;\alpha^{ice}$$
  (5) $$S_{12}^{ice}\;(b)=2{TL}^{air}+2{St}^{ice}+10.5\;\times \;\alpha^{ice}$$
  (6) $$S_{12}^{ice}\;(c)=2{TL}^{air}+2{St}^{ice}+12\;\times \;\alpha^{ice}$$
  where *TL^air^* = 0.5 × $S_{12}^{air}$, and *TL^lq^* and *TL^ice^* in dB are the transduction losses of the input and output transducers (assumed to be identical) measured, respectively, without loading (*air*), with water (*lq*), and with ice (*ice*); *St^lq^* and *St^ice^* in dB are the losses produced by scattering the wave at the plate/liquid and plate/ice steps (assumed identical to the liquid/plate and ice/plate steps); *α^lq^* and *α^ice^* in dB/mm are the attenuation coefficients of a mode with water and ice loadings (assumed to be much larger than for air), respectively; the propagation paths coated with water or ice in the delay lines, as shown in Figure 2a–c, are 22.5, 10.5, and 12 mm, respectively. The numbers of linear equations and unknown parameters {*TL^lq^*, *TL^ice^*, *St^lq^*, *St^ice^*, *α^lq^*, *α^ice^*} are equal to 6, so the solution of the system (1)–(6) is unambiguous. The precision of the solutions was about ±10%.

Equations (1)–(6) allow estimate partial contributions to the ice responses $\Delta S_{12}=S_{12}^{ice}-S_{12}^{lq}$ for each mode, test sample, and delay line configuration. They also permit separate transduction effects from that of propagation as well as comparing transducer efficiency for different conditions on plate faces (air, liquid, ice) without knowledge of the explicit dependence of the transduction on the coupling constant of the wave and electric impedance of the transducer. However, this approach is unable to discriminate between different attenuation mechanisms because it integrates all available contributions, such as the wave radiation into the test liquid, wave radiation into ice, viscoelastic loss in liquid and ice, wave scattering from ice crystallites, etc.

An analysis of the Equations (1)–(6) from the measured $S_{12}^{lq}$ (a), $S_{12}^{lq}$ (b), $S_{12}^{lq}$ (c), $S_{12}^{ice}$ (a), $S_{12}^{ice}$ (b), and $S_{12}^{ice}$ (c) is accomplished for the Y,Z-LiNbO_3_ plate, water layer, and ice layer of the same normalized thickness *h/λ*, as an example.

Finally, in order to clarify the experimental results, the changes in the mode characteristics were numerically calculated for plates without loading (free faces) and for the same plates with water or ice loadings on one face. Calculations were accomplished using well-approved software [40] and theoretical methods described earlier in detail [41]. Material constants for the LiNbO_3_ plate at room temperature were taken from [42]. Normalized thickness of the plate, water layer and ice layer are taken equal to that of experimental (*h*/*λ* = 1.67). The orders of the modes in different structures are thoroughly controlled by small variations of *h*/*λ* from 0 (uncoated plate) to final value *h*/*λ* = 1.67 (test structure) resulting to step by step variations in phase velocities, coupling constants, and elastic displacements of the modes.

**Figure 3:**
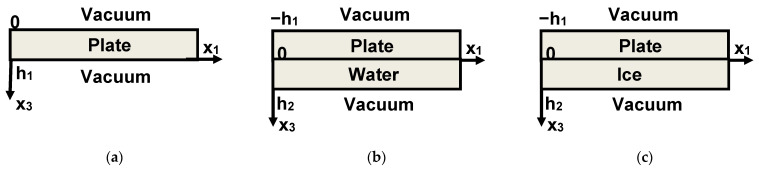
Geometries of the structures (**a**) “vacuum-Y,Z LiNbO_3_ plate-vacuum”, (**b**) “vacuum-Y,Z LiNbO_3_ plate—water layer—vacuum”, (**c**) “vacuum-Y,Z LiNbO_3_ plate—ice layer—vacuum” considered theoretically.

It should be remembered that for numerical calculations at different temperatures the temperature variations of the material constants of the plate, water, and ice should be taken into account. The elastic, piezoelectric, and dielectric constants and the density of the piezoelectric material at actual temperature *T* were written as [43,44]. The temperature coefficients for LiNbO_3_ were taken from [43]. Material constants of water and ice at different temperatures were taken from [45,46,47,48]. The coefficient of the thermal expansion for ice (0.5 × 10^−4^ C^−1^) and the thermal variations of the ice dielectric constant (11 × 10^−4^ C^−1^) at *T* > −35 °C were taken from [45]. In our calculations, water was considered as nonviscous and nonconductive liquid. We have theoretically considered three structures with geometries presented in Figure 3.

## 3. Results and Discussion

**Figure 4:**
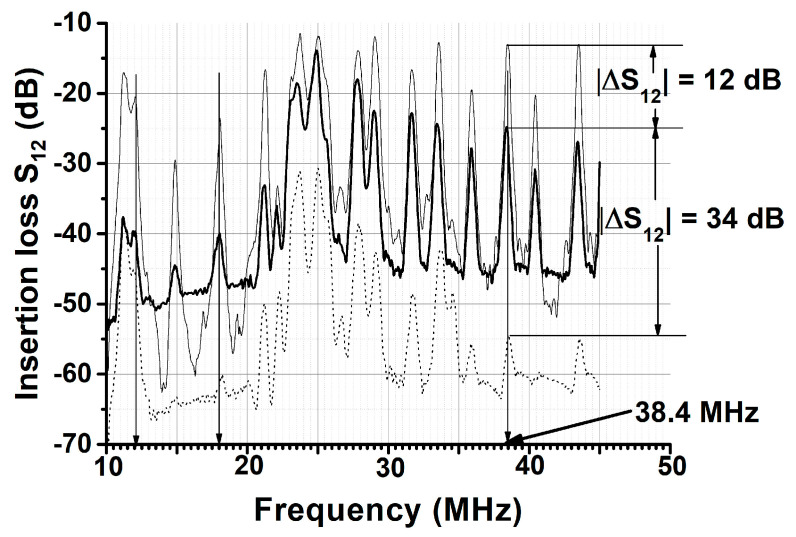
Insertion loss *S*_12_ of the delay line implemented on the Y,Z-LiNbO_3_ plate with a normalized thickness of *h/λ* = 1.67, as measured by the network analyzer in air at 20 °C (thin solid), with water loading at 20 °C (thick solid), and ice loading at −15 °C (dotted). The position of the water (ice) sample on the plate is shown in Figure 2a. The velocity and electromechanical coupling coefficient of the mode at 38.4 MHz are *V_n_* = 11,563 m/s and $k_{n}^{2}$ = 1.12%.

**Figure 5:**
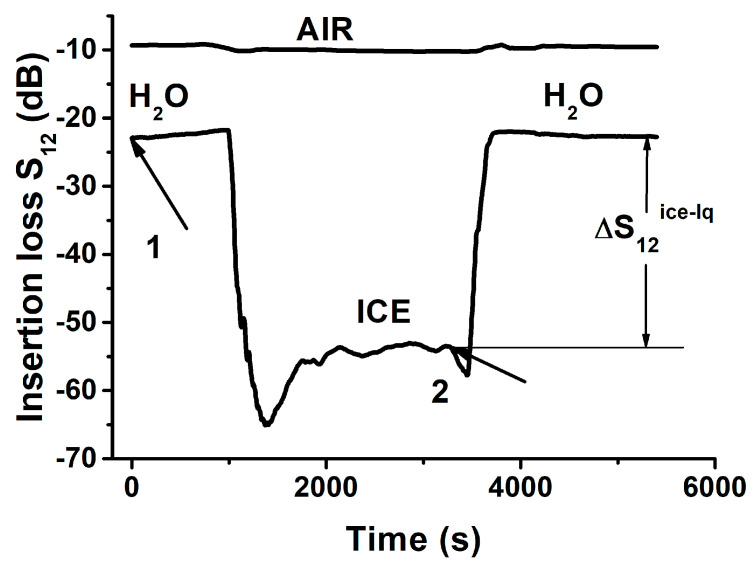
Time variation of the insertion loss *S*_21_ of the delay line coated with distilled water measured for *jump* cooling (from +20 °C to −15 °C, arrow 1) and *jump* heating (from −15 °C to +20 °C, arrow 2). AIR: the measurements without liquid. H_2_O-ICE-H_2_O: the measurements with water and ice. Plate: Y,Z-LiNbO_3_, *h*/*λ* = 1.67. Mode: *f_n_* = 38.4 MHz. The position of the water (ice) sample on the plate is shown in Figure 2a.

**Figure 6:**
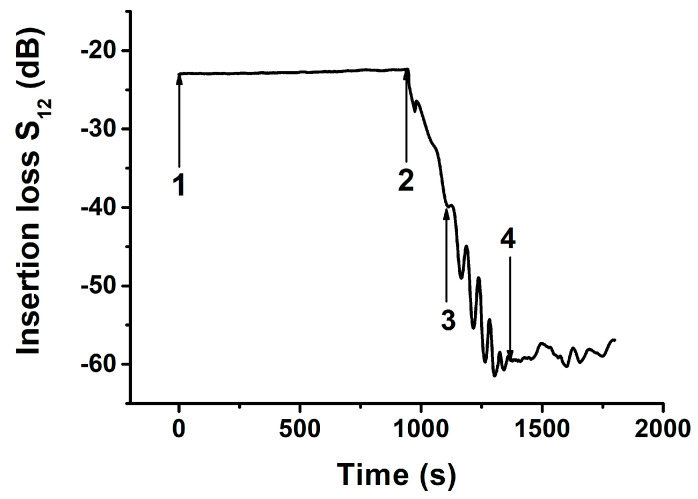
Time variation of the insertion loss *S*_12_ of the delay line coated with distilled water measured for fluent cooling from +20 °C to −15 °C in intervals of 5 °C with a pause of 60 s at each temperature. 1—The start of the cooling, 2—The start of the ice formation (−5 °C, 900 s), 2, 3, 4—water-ice two-phase system (from −5 °C to −15 °C, 900–1300 s), 4-homogeneous ice phase (−15 °C, >1300 s). Y,Z-LiNbO_3_ plate, *h*/*λ* = 1.67. Mode: *f_n_* = 38.4 MHz. The position of the water (ice) sample on the plate is shown in Figure 2a.

**Figure 7:**
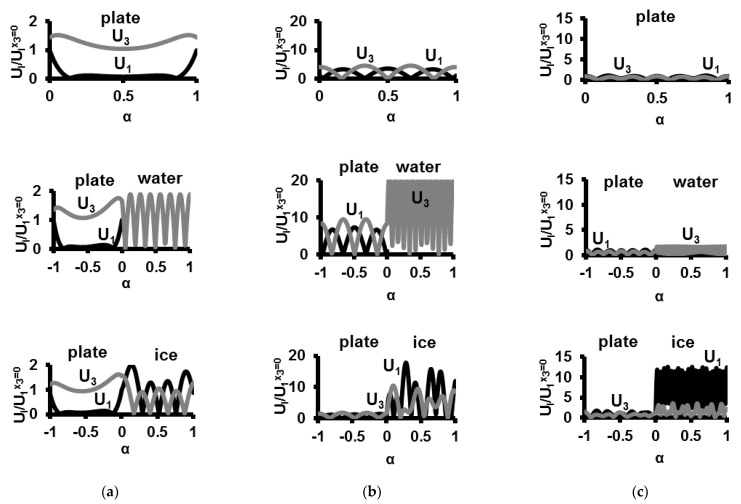
The profiles of the normalized mechanical displacements of the waves vs thickness x_3_ = α×h/λ of the structures calculated for uncoated Y,Z-LiNbO_3_ plate (upper row), “Y,Z-LiNbO_3_ plate—water layer” structure (middle row), and “Y,Z-LiNbO_3_ plate—ice layer” (lower row) structure. The wave frequencies are 11.4 MHz (**a**), 18.05 MHz (**b**), and 38.4 MHz (**c**). The thickness of the plate and layers are *h*/*λ* = 1.67.

The results of the measurements are presented in Figure 4, Figure 5 and Figure 6 and Table 1 and Table 2. The results of the numerical calculations of the acoustic waves under the study phase velocities for electrically open *V_n_* and shorted $V_{n}^{m}$ structures, the electromechanical coupling coefficient $k_{n}^{2}$, and the vertical displacement component $U_{3}/U_{1}^{x_{3}=0}$ are given in Table 2. The normalized displacement components $U_{1}/U_{1}^{x_{3}=0}$ and $U_{3}/U_{1}^{x_{3}=0}$ through the structure thickness for waves under study are presented in Figure 7.

Figure 4 shows the typical frequency dependence of the insertion loss *S*_12_ for acoustic plate modes detected at different frequencies in the plate with free faces (thin solid), with water loading (thick solid), and with ice loading (dotted). Most modes belong to the Lamb family, but some of them may be quasi-longitudinal (e.g., in ST,X-quartz) or quasi-shear-horizontal (e.g., in ST,X+ 90°-quartz). As usual, the frequencies of the modes *f_n_* were determined by the phase velocity *V_n_* and wavelength *λ* (*f_n_ = V_n_/λ*); the amplitudes of the modes depend on the electromechanical coupling coefficients $k_{n}^{2}$ and the attenuation: the more coupling and less attenuation there is, the larger the amplitude in general. As can be seen, all modes in Figure 4 have different correlations between the insertion loss measured in air $S_{12}^{air}$, with water $S_{12}^{lq}$, and with ice $S_{12}^{ice}$, but the ration $S_{12}^{air}$ < $S_{12}^{lq}$ < $S_{12}^{ice}$ is valid every time. For example, the mode 11.4 MHz propagating in the Y,Z-LiNbO_3_ plate with a normalized thickness of *h/λ* = 1.67 has $S_{12}^{air}$ << $S_{12}^{lq}$ and $S_{12}^{lq}$ ≤ $S_{12}^{ice}$. On the other hand, the modes 18.05 and 38.4 MHz in the same plate have much higher $S_{12}^{lq}$ losses than those for air, as well as $S_{12}^{air}$ and $S_{12}^{ice}$ losses much higher than those for water $S_{12}^{lq}$. The response $\Delta S_{12}^{ice-lq}$ of the mode 38.4 MHz towards water-to-ice transformation (34 dB) is the largest among all ice responses measured so far using other waves and plates (Table 1).

Figure 5 shows the time variation in the insertion loss of the best mode when the temperature of the delay line was changed in a step from +20 °C to −15 °C (arrow 1) and from −15 °C to +20 °C (arrow 2). This shows that when the plate has no liquid (AIR) the temperature variations in *S*_12_ loss are very small (<1 dB), as expected. On the other hand, when the plate is coated with water, the same variations are small again, but only at the beginning, when the water is still liquid. As the temperature drops (−5 °C, 900 s), the liquid starts its transformation to a solid phase, and the *S_12_* value drops sharply. Maximal change $\Delta S_{12}^{ice-lq}$= 34 dB is attributed to the total transformation of one substance to the other, when ice, growing from its outside, approaches the water–plate interface. Small variations in *S_12_* at its maximum (1800 ÷ 3200 s, Figure 5) indicate that at *T* = −15 °C the ice sample is homogeneous, contains no water, and has rigid contact with the plate surface. Similarly, when the ice/plate structure was heated, the loss *S_12_* started to decrease almost immediately. This jump may be interpreted as the appearance of a thin water layer at the ice–plate interface. Finally, when the *S*_12_ approached the initial value and became stable again, the ice sample transformed to water completely, and the process was completed.

Figure 6 shows details of the time variations of the insertion loss *S*_12_, when liquid (water) cooled fluently from +20 °C to −15 °C in intervals of 5 °C with pauses of 60 s at each temperature. It is seen that, in addition to a stable part at the beginning and a fast decrease as the temperature decreases (−5 °C, 900 s, as seen in Figure 5), the *S*_12_ parameter moved up and down for intermediate temperatures (−5 °C, arrow 2 to −15 °C, arrow 4). This peculiarity, in our minds, indicates that the icing process is not instantaneous. We believe that the appearance of the picks originates from the simultaneous existence of two water/ice phases with small ice crystallites in the liquid broth.

However, it is still unclear why the start of the ice formation (−5 °C), the existence of the two-phase system (−5 °C to −15 °C), and the formation of homogeneous ice (−15 °C) were detected at lower temperatures than may be expected. This fact demands additional investigations using, for example, the visual control of a liquid–solid transformation together with its acoustic analysis.

Analysis Equations (1)–(6) based on the measured $S_{12}^{lq}$ (a), $S_{12}^{lq}$ (b), $S_{12}^{lq}$ (c), $S_{12}^{ice}$ (a), $S_{12}^{ice}$ (b), and $S_{12}^{ice}$ (c) were accomplished for modes 11.4, 18.05, and 38.4 MHz, as an example (Table 2). As expected, results of the estimations correlate with data calculated numerically: the more the normal displacement of the wave $U_{3}^{pl-lq}$ (line 9), the more the wave radiation into the liquid and the higher the attenuation coefficient *α^lq^* (line 4); the more coupling constants there are for $k_{n}^{2}$ (lq) (line 12) and $k_{n}^{2}$ (ice) (line 13), the less the transduction loss is for *TL^lq^* (line 5) and *TL^ice^* (line 7). Furthermore, (i) for mode 11.4 MHz the integral value of the insertion loss and the partial contributions to the value, which are attributed to attenuation *α*, transduction TL, and steps St, are almost the same both for water and ice. Therefore, the mode is insensitive towards ice formation according to this experiment shown in Figure 4, *f* = 11.4 MHz, (ii) on the contrary, for mode 38.4 MHz, the dominant contribution is attributed to attenuation and the attenuation coefficient for ice *α^ice^* (line 6) is four times higher than it is for water *α^lq^* (line 4). Therefore, the mode 38.4 MHz has high water-to-ice sensitivity, in agreement with the experiment shown in Figure 4, *f* = 38.4 MHz, (iii) and in contrast to mode 38.4 MHz, the dominant response for mode 18.05 MHz originated from transduction loss, increasing from 6.8 dB for water (line 5) to 12.2 dB for ice (line 7), in accordance with decreasing the relevant coupling constants $k_{n}^{2}$ (lq) (line 12) and $k_{n}^{2}$ (ice) (line 13).

*S*_12_*^air^*, *S*_12_*^lq^*, *S*_12_*^ice^*are the insertion losses of the delay lines measured without loadings for air, and with loadings for water and ice; *α^lq^*, *α^ice^*, *TL^lq^*, *TL^ice^* are the attenuation coefficients and transduction losses estimated from Equations (1)–(6); *k_n_*^2^ (air), *k_n_*^2^ (lq), *k_n_*^2^ (ice) are the coupling constants of the modes calculated numerically for the plate without loading, with water, and with ice for metallization on the free bottom of the plate (Figure 2). *U*_3_*^pl^*/*U*_1_*^pl^*, *U*_3_*^pl-lq^*/*U*_1_*^pl^*, *U*_3_*^pl-ice^*/*U*_1_*^ple^* are the vertical displacements of the modes calculated numerically on the free face of the plate; the water/plate and ice/plate interfaces are normalized to the relevant longitudinal displacements *U*_1_*^pl^* at boundary *x*_3_ = 0 (Figure 3). $V_{n}^{pl}$, $V_{n}^{pl-lq}$, $V_{n}^{pl-ice}$ are the numerically calculated phase velocities of the corresponding waves. $V_{n}^{pl_{m}}$, $V_{n}^{{\left(pl-lq\right)}_{m}}$, $V_{n}^{{\left(pl-ice\right)}_{m}}$ are the numerically calculated phase velocities of the same waves for the electrically shorted structure at *x*_3_ = 0 (Figure 3).

In general, the calculations show that amount of modes existing in water/plate and ice/plate structures are much larger than that is in uncoated plate of the same thickness. For example, for the thickness of the plate, water, and ice equal to *h*/*λ* = 1.67 (experimental value) the difference in velocities of the neighbor modes is about 1000 m/s in uncoated plate, while it is about 200 m/s in the plate coated with water or ice. Velocities and electro-mechanical coupling constants of the modes in layered structures reduce with thickness of the layers. The values of these constants for most modes in the structures are very small except for the modes originated from relevant modes in uncoated plate.

The profiles of the normalized mechanical displacements of the modes vs thickness *x*_3_ = *α* × *h*/*λ* of uncoated plate, the plate coated with water, and the same plate coated with ice are presented in Figure 7.

Some results of the numerical calculations (Figure 7) correlate with experimental data (Table 2) presented for delay line structure Figure 2b, when transduction loss of the input and output transducers are eliminated from consideration: (i) in contrast to mode 38.4 MHz the modes 18.05 and 11.4 MHz have dominant *U*_3_ displacement in water. As a result, the radiation losses of the modes are 4.9, 13.7, and 19 dB, respectively, (ii) in contrast to mode 11.4 MHz the modes 18.05 MHz and 38.4 MHz have larger displacements *U*_1_ and *U*_3_ in ice. As a results, the icing responses of the modes *S*_12_*^ice^* − *S^lq^* are 0.8, 13.7, and 19.7 dB, respectively, (iii) all displacements and, hence, elastic energy of the mode 38.4 MHz are strongly concentrated into the ice layer. As a result, the mode has largest icing response as compared with other two modes.

On the whole, the calculations showed that the number of acoustic waves existing in the ice–plate double-material structure is much higher than for uncoated plates in the same frequency range. This result may be attributed to supplementary wave reflections from additional boundaries in the ice–plate double-material structure.

Compared to the uncoated plate, the phase velocities of the Lamb waves are not decreased too much due to water and ice loadings. This may be explained by the small acoustic impedance of the loading materials.

When the temperature is decreased, velocities of the acoustic waves both in the uncoated Y,Z LiNbO_3_ plate and in the plate–ice layered structure are increased. This result is explained by a stronger increase in effective elastic modulus and a lower increase in the density of the plate and ice at lower temperatures.

On the contrary, the phase velocities of the acoustic waves in the plate–water layered structure are decreased with decreasing temperature. Here, the temperature decrease of the velocity in water is much higher than the temperature increase of the velocity in the plate.

## 4. Conclusions

Most Lamb waves are able to propagate along piezoelectric plates coated with liquid (water) and solid (ice) layers, though the layers produce additional propagation, transduction, and radiation losses. As compared with uncoated plates, the number of waves existing in their coated counterparts is increased, the velocities of the waves are decreased, and the wave losses for water are usually less than those for ice. An increase in the losses allows the detection of the water-to-ice transformation using acoustic wave propagation in plates. When the water/plate structure is cooled and the losses are increased, the stabilization of the losses at their maximum value may be attributed to the total transformation of water substance to ice. When the ice/plate structure is heated and the ice is melted, the decrease in the losses may be interpreted as the appearance of a thin water layer at the ice–plate interface. When losses move up and down at intermediate temperatures, the existence of two (water and ice) phases may be supposed. As a result, in order to transform water to ice completely, the water sample should not simply be cooled below a definite temperature (as for most liquids) but must be cooled within a certain temperature interval from about −5 to −15 °C. All of these details may turn out to be useful for some applications where the control of ice formation is an important problem (aircraft wings, ship bodies, roads, etc.).

## Figures and Tables

**Table 1:** The modes with the largest acoustic wave ice responses measured in different plates with the same normalized thickness *h*/*λ* = 1.67.

Plate Material	*f_n_*, MHz	$\left|\Delta S_{12}^{lq-air}\right|,\;\mathrm{dB}$	$\left|\Delta S_{12}^{ice-lq}\right|,\;\mathrm{dB}$
Y,Z-LiNbO_3_	38.4	11	34
Y,Z+ 90°-LiNbO_3_	41.21	2.7	29
ST,X+ 90°-SiO_2_	20	0.3	20
ST,X-SiO_2_	19	0.2	9
36°Y, X+ 90°-LiTaO_3_	27.5	5.3	8
36°Y,X-LiTaO_3_	23	3.5	16

**Table 2:** Comparison of the estimations deduced from Equations (1)–(6) with the mode properties calculated numerically. Normalized thickness of the Y,Z-LiNbO_3_ plate, water layer, and ice layer are *h*/*λ* = 1.67.

#	Parameter and Delay Line Configuration	*f_n_* = 11.4 MHz	*f_n_* = 18.05 MHz	*f_n_* = 38.4 MHz
1	$\left|S_{12}^{air}\right|$, dB	9.4	17.0	11.4
2	$\left|S_{12}^{lq}\right|$, dB (Figure 2a)	35.1	41.4	22.0
	$\left|S_{12}^{lq}\right|$, dB (Figure 2b)	28.4	30.7	16.3
	$\left|S_{12}^{lq}\right|$, dB (Figure 2c)	22.0	29.2	16.9
3	$\left|S_{12}^{ice}\right|$, dB (Figure 2a)	37.1	57.8	56.0
	$\left|S_{12}^{ice}\right|$, dB (Figure 2b)	29.2	44.4	36.0
	$\left|S_{12}^{ice}\right|$, dB (Figure 2c)	23.5	54.0	41.0
4	*α^lq^*, dB/mm	1.5	1.75	0.5
5	TL*^lq^*, dB	3.5	6.8	5.8
6	*α^ice^*, dB/mm	1.5	1.5	2
7	TL*^ice^*, dB	6	12.2	7
8	$U_{3}^{pl}/U_{1}^{pl_{}(x_{3}=0)}$ at *T* = 23 °C	1.45	3.95	0.85
9	$U_{3}^{pl-lq}/U_{1}^{pl_{}(x_{3}=0)}$ at *T* = 23 °C	1.67	8.3	0.82
10	$U_{3}^{pl-ice}/U_{1}^{pl_{}(x_{3}=0)}$ at *T* = −13 °C	1.55	1.7	1.1
11	$k_{n}^{2}$ (air), % at *T* = 23 °C	3.9	0.3	1.12
12	$k_{n}^{2}$ (lq), % at *T* = 23 °C	2.9	0.16	0.9
13	$k_{n}^{2}$ (ice), % at *T* = −13 °C	3.1	0.01	0.06
14	$V_{n}^{pl}/V_{n}^{pl_{m}}$ at *T* = 23 °C	3450/3381	5375/5366	11,563/11,498
15	$V_{n}^{pl-lq}/V_{n}^{{\left(pl-lq\right)}_{m}}$ at *T* = 23 °C	3424/3373	5330/5325	11,496/11,444
16	$V_{n}^{pl-ice}/V_{n}^{{\left(pl-ice\right)}_{m}}$ at *T* = −13 °C	3442/3388	5215/5214	11,391/11,386
